# Annual of the Universal Medical Sciences

**Published:** 1889-12

**Authors:** 


*Annual of the Universal Medical Sciences.* Edited by C. E. SAJOUS, M.D. Five Volumes. Philadelphia, New York, and London: F. A. DAVIS. 1889.

We have already commented in the most favourable terms on the excellence of this magnificent *Annual.* The issue for 1889 is an improvement on that for 1888, both in regard to the remarkable completeness of the index, and in the improved facilities for reference to journals quoted. The work has no rival in the English language. At the very moderate price at which it is issued, it ought to be in the hands of every English practitioner of our art.

